# Evaluation of the Implementation and Effectiveness of Community-Based Brain-Computer Interface Cognitive Group Training in Healthy Community-Dwelling Older Adults: Randomized Controlled Implementation Trial

**DOI:** 10.2196/25462

**Published:** 2021-04-27

**Authors:** Pei Shi Yeo, Tu Ngoc Nguyen, Mary Pei Ern Ng, Robin Wai Munn Choo, Philip Lin Kiat Yap, Tze Pin Ng, Shiou Liang Wee

**Affiliations:** 1 Geriatric Education and Research Institute Singapore Singapore; 2 Khoo Teck Puat Hospital Singapore Singapore; 3 National University of Singapore Singapore Singapore

**Keywords:** group-based computerized cognitive training, cognition, gait, community program implementation, healthy older adults, cognitive, community program, cognitive training, elderly, aging

## Abstract

**Background:**

Cognitive training can improve cognition in healthy older adults.

**Objective:**

The objectives are to evaluate the implementation of community-based computerized cognitive training (CCT) and its effectiveness on cognition, gait, and balance in healthy older adults.

**Methods:**

A single-blind randomized controlled trial with baseline and follow-up assessments was conducted at two community centers in Singapore. Healthy community-dwelling adults aged 55 years and older participated in a 10-week CCT program with 2-hour instructor-led group classes twice a week. Participants used a mobile app to play games targeting attention, memory, decision making, visuospatial abilities, and cognitive flexibility. Implementation was assessed at the participant, provider, and community level (eg, reach, implementation, and facilitators and barriers). Effectiveness measures were the Repeatable Battery for the Assessment of Neuropsychological Status (RBANS), Color Trails Test 2 (CTT-2), Berg Balance Scale, and GAITRite walkway measures (single and dual task gait speed, dual task cost, and single and dual task gait variability index [GVI]).

**Results:**

A total of 94 healthy community-dwelling adults participated in the CCT program (mean age 68.8 [SD 6.3] years). Implementation measures revealed high reach (125/155, 80.6%) and moderate adherence but poor penetration of sedentary older adults (43/125, 34.4%). The effectiveness data were based on intention-to-treat (ITT) and per-protocol (PP) analysis. In the ITT analysis, single task GVI increased (*b*=2.32, *P*=.02, 95% CI [0.30 to 4.35]) and RBANS list recognition subtest deteriorated (*b*=–0.57, *P*=.01, 95% CI [–1.00 to –0.14]) in both groups. In the PP analysis, time taken to complete CTT-2 (*b*=–13.5, *P*=.01, 95% CI [–23.95 to –3.14]; Cohen *d* effect size = 0.285) was faster in the intervention group. Single task gait speed was not statistically significantly maintained in the intervention group (*b*=5.38, *P*=.06, 95% CI [–0.30 to 11.36]) and declined in the control group (Cohen *d* effect size = 0.414). PP analyses also showed interaction terms for RBANS list recall subtest (*b*=–0.36, *P*=.08, 95% CI [–0.75 to 0.04]) and visuospatial domain (*b*=0.46, *P*=.08, 95% CI [–0.05 to 0.96]) that were not statistically significant.

**Conclusions:**

CCT can be implemented in community settings to improve attention and executive function among healthy older adults. Findings help to identify suitable healthy aging programs that can be implemented on a larger scale within communities.

**Trial Registration:**

ClinicalTrials.gov NCT04439591; https://clinicaltrials.gov/ct2/show/NCT04439591

## Introduction

Cognitive functions decline with age but can be maintained or improved through training [1,2]. Cognitive training involves structured, frequent, and repeated engagement in standardized cognitively demanding tasks targeting specific cognitive domains [3]. In old age, the brain still possesses neural plasticity, or the lifelong ability for physical and functional change in response to sensing, perceiving, and learning. Cognitive training can stimulate neuroplasticity and increase cognitive reserve in older adults [4]. Cognitive stimulation results in molecular, synaptic, and neural alterations in animal brains [5]. In humans, cognitive training increased serum brain-derived neurotropic factor, which plays an important role in memory processing [6]. Brain imaging post training revealed changes in brain activity during task performance, along with long-term global changes [7].

Technological advancements have encouraged the rise of computerized cognitive training (CCT). A meta-analysis concluded that CCT was modestly effective at improving cognitive performance in healthy older adults [8]. However, efficacy varied across cognitive domains and was largely determined by the study designs. Small to moderate effect sizes were found for verbal and nonverbal memory, working memory, processing speed, and visuospatial abilities but not for executive function and attention [8]. The same study also found that group-based training was more effective than home-based training with limited benefit when training more than 3 times per week, potentially due to the presence of direct supervision by a trainer in a group-based training to ensure adherence, treatment fidelity, compliance, and motivational support to master challenging tasks that are otherwise easy to avoid [8]. Another review uncovered improvements in everyday functioning and neuropsychological tests in untrained tasks [1]. Cognitive processing also plays an important role in balance and gait and reduced cognitive processing speed is a contributing factor to falls in older adults. This relationship may be explained by the fact that higher order cognitive functions (eg, executive functions) are called upon while walking. Attention and executive functions are associated with mobility, and several studies have shown that CCT improved gait speed and balance, with greater efficacy for sedentary older adults with low gait speed [9-12].

Other than an online study in healthy older adults [13], most studies have evaluated the efficacy of CCT on cognition and real-world function in healthy adults in research settings; there have been few implementation studies in real-world settings. Sufficient evidence exists for research to move beyond laboratory trials to evaluate CCT implementation and effectiveness in community-based settings [1].

In Singapore, two laboratory trials improved cognitive function in healthy older adults but showed inconsistent efficacy across cognitive domains [14,15]. These studies used the developmental version of a brain-computer interface cognitive training program, which has been commercialized (NeeuroFIT) and can be delivered as a community-based CCT program.

Therefore, in this study we aim to evaluate (1) the implementation of this community-based CCT at the participant, provider, and community level and (2) its effectiveness on cognition, gait, and balance in healthy older adults. We followed the approach to cognitive training targeting attention, memory, decision making, visuospatial abilities, and cognitive flexibility developed and implemented by the developer of the cognitive training program.

## Methods

### Setting

Community centers are neighborhood public spaces designed to provide sports, enrichment programs, and amenities. Community centers are equipped with the facilities and administrative processes that would support community-based CCT implementation and would be suitable sites for eventual wider community adoption of CCT. The CCT developer (Neeuro Pte Ltd) partnered with two community centers to offer the program at a subsidized rate with aid from a social enterprise grant.

### Participants

Healthy community-dwelling adults aged 55 years and older participated in the CCT between September 2017 and November 2018. We targeted 55 years and above as that is the age for withdrawal of retirement funds from the national savings scheme in Singapore. As some may choose to retire at this age, there is demand for health-related activities. Recruitment was conducted at two community centers in western Singapore. The study initially sought to recruit sedentary older adults who exercised less than once per week. However, most participants at the first community center were not sedentary. As such, participants at both community centers were recruited regardless of their exercise frequency. The exclusion criteria were: (1) unable to understand English or Mandarin, (2) cognitive impairment (ie, modified Mini-Mental State Examination score ≤23), (3) diagnosis of neuropsychiatric disorder(s), (4) ongoing use of psychotropic medications, (5) depression (ie, Geriatric Depression Scale >9), (6) severe walking or balance impairments (eg, wheelchair-dependent), (7) self-reported vertigo, (8) visual acuity <20/80, (9) color-blind, (10) participation in a cognitive training program within the past year, or (11) plans for a balance training program during the study period. The study was conducted in accordance with the Declaration of Helsinki and received ethics approval from the National Healthcare Group’s Domain Specific Review Board. Written informed consent was obtained from all participants prior to study enrollment.

### Intervention

The 10-week CCT program (NeeuroFIT) consisted of 2-hour instructor-led group classes conducted twice a week in English or bilingually (ie, English and Mandarin). Trained instructors guided participants through game-based training targeting attention, memory, decision making, visuospatial abilities, and cognitive flexibility using a mobile app (Memorie, Neeuro Pte Ltd). CCT was gamified to enable sustained interest and facilitate adaptive training where participants progressed to more cognitively demanding levels. Selected games were paired with an electroencephalography headset (Senzeband, Neeuro Pte Ltd) that quantified users’ attention into scores that influenced their in-game avatar control or game performance. Participants paid a subsidized fee of SGD $20 (US $15) for the CCT.

### Procedure

**Figure 1:**
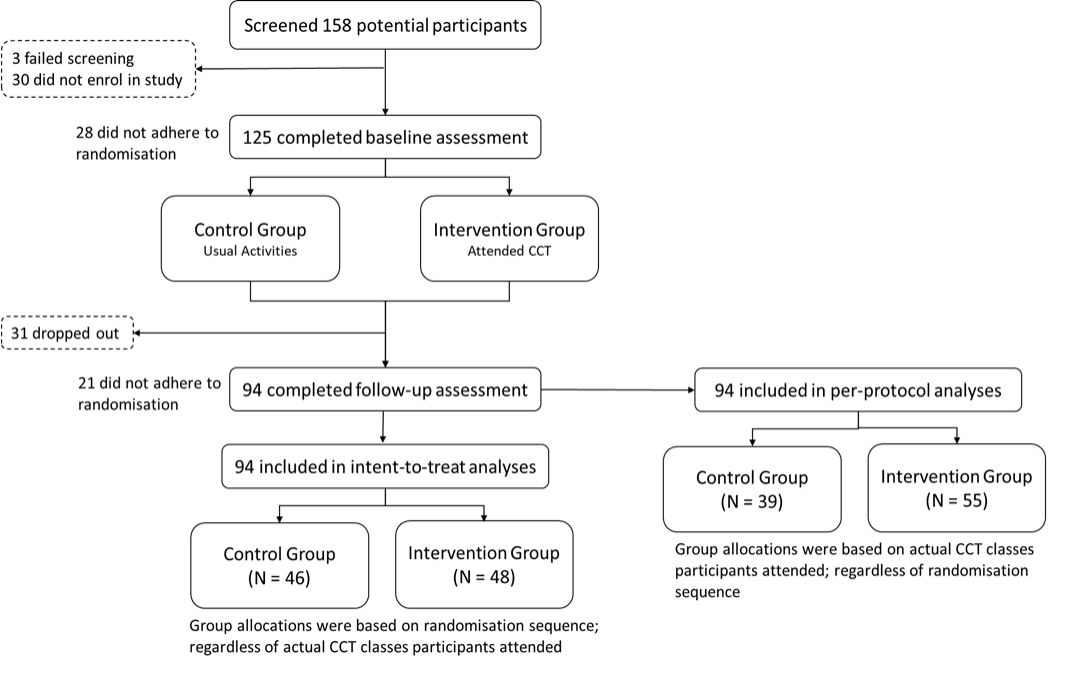
Participant flow diagram. CCT: computerized cognitive training.

Within each community center, participants were randomized into the intervention or waitlist control group. Randomization sequence for the first community center was generated using Excel (Microsoft Corp) by a CCT provider staff member with no participant contact. Randomization sequence for the second community center was generated via a web-based randomization service by a biostatistician without participant contact [16]. A single-blind randomized controlled trial (RCT) design with baseline and follow-up assessment was used (Figure 1). Participants were blinded in this study. The control group continued with their usual activities while the intervention group attended CCT. Assessments were completed within 2 weeks of the CCT.

### Outcome Measures

#### Implementation Measures

Implementation was assessed at the participant, provider, and community level (Multimedia Appendix 1) [17]. The table describes measures taken at each of the levels and the type of data collected.

#### Effectiveness Measures

Assessments were conducted in English or Mandarin by trained study team members blinded to group allocation. Participants completed the Color Trails Test 2 (CTT-2) prior to the Repeatable Battery for the Assessment of Neuropsychological Status (RBANS) to prevent fatigue induced by RBANS from influencing CTT-2 performance. The order of other assessments was not fixed. Forms A and B of the CTT-2 and RBANS were used for baseline and follow-up assessments, respectively, to minimize learning effects. Standardized test instructions were translated from English to Mandarin for Mandarin-speaking participants.

The CTT-2 assessed sustained visual attention, executive function, and visuomotor skills [18]. This test possesses test-retest reliability and has convergent validity with the Trail Making Test, which indexes attention, visuospatial abilities, and cognitive flexibility [18]. As compared with the Trail Making Test, CTT-2’s use of colors instead of letters renders it more cross-culturally appropriate, especially in the current sample that consists of both English and Mandarin speakers.

RBANS, the primary effectiveness outcome measure, is a neuropsychological battery comprising 12 subtests assessing 5 cognitive domains: immediate memory, visuospatial, language, attention, and delayed memory. It is reliable and has been validated against other tests assessing corresponding cognitive domains [18-20]. This study used RBANS Form A modified by the Singapore Longitudinal Aging Studies research team for Singaporean older adults [21]. To minimize differences in test difficulty across forms, subtest scores were standardized within each assessment (ie, across groups) before being summed to derive the domain scores and total score. Given inconsistencies in prior studies [14,15], analyses would assess changes in RBANS total score, domain scores, and subtest scores.

The Berg Balance Scale assesses balance via performance on 14 functional tasks [22]. Performance for each task was scored independently using a 0 to 4 scale, with higher scores reflecting better balance. Individual task scores were summed to derive a composite score for analyses.

Gait assessments were conducted using the 6 meter GAITRite walkway [22]. Participants started 1 meter behind the walkway and walked to a chair placed 1 meter past the end of the walkway. Under the single task condition, participants walked at their habitual walking speed. Under the dual task condition, participants walked while performing serial sevens subtraction. Participants were not instructed to prioritize either the cognitive or walking task. After attempting the dual task, participants completed serial sevens subtraction while seated. Participants completed 1 practice trial and 3 actual trials for each condition. Calculated using the mean of the actual trials, gait parameters examined in analyses included (1) single task gait speed (cm/s), (2) dual task gait speed (cm/s), (3) dual task cost, (4) single task gait variability index (GVI), and (5) dual task GVI. Dual task cost was derived using

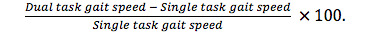



GVI was calculated as in Gouelle et al [23].

### Statistical Analyses

Some participants did not adhere to the randomization sequence (Figure 1). As such, 2 sets of parallel analyses were conducted. Intention-to-treat (ITT) analyses were based on the randomization sequence regardless of actual CCT class attendance. Per-protocol (PP) analyses grouped participants based on the CCT classes they attended regardless of the randomization sequence. Baseline group differences were examined using Pearson chi-square tests for categorical data, independent samples *t* tests for parametric data, and Mann-Whitney *U* tests for nonparametric data. Changes in outcome variables were explored using mixed models with missing data handling. Missing data were assumed to be missing at random. This assumption was made when dummy variables were created for missing variables (1 as missing; 0 as observed), and *t* tests and chi-square tests were performed between these dummy variables and other variables. The missingness on these variables was found to be related to the values of other variables. Parametric outcome variables were estimated using maximum likelihood with robust standard errors, while nonparametric data were assumed to be right censored and were estimated using maximum likelihood. A 2-sided *P* value of .05 was deemed statistically significant; no adjustments were made despite multiple comparisons for RBANS due to the explorative nature of these analyses. Statistical analyses were completed using R (version 3.4.4, R Foundation for Statistical Computing) and MPlus (version 7.11, UCLA Statistical Consulting Group).

## Results

### Implementation Measures: Participant Level

Of 155 individuals screened, 125 agreed to participate and completed a baseline assessment indicating reach as 80.6% (Figure 1). Moderate adherence was observed. First, 22.4% (28/125) of participants did not adhere to the randomization sequence (eg, due to conflicting schedules). Of these, 64% (18/28) attended CCT classes for the intervention group despite being randomized to the control group. The remaining 10 switched from intervention group to the control group. Second, the dropout rate was 24.8% (31/125) with no observed group differences in ITT (*P*=.38; Multimedia Appendix 2) or PP analyses (*P*=.20; Table 1). Third, participants attended 80% (16/20) of classes. Class attendance for the intervention group was higher than the control group in ITT (*P*=.02; Multimedia Appendix 2) and PP analyses (*P*=.03, Table 1).

Facilitators and barriers to CCT participation were obtained from participant feedback and observations by the study team. CCT classes, the mobile app, and electroencephalography headset were well received. Trainers’ in-class guidance and encouragement motivated participants to continue with classes and increasingly cognitively demanding games. A few expressed interests in more advanced level CCT. Barriers to program participation included busy schedules, loss of interest in CCT, and loss to follow-up. A few cited fatigue from prolonged screen use, noisy class environment, difficulty in selected games, and belief that CCT was ineffective. Two control group participants from the first community center dropped out from follow-up assessment and CCT as they did not receive reminders regarding CCT commencement. The program provider ensured that all participants from the second community center were reminded prior to CCT. Program participation was also influenced by proximity to implementation sites and strength of community ties. Most participants from the first community center lived near the community center and were well acquainted with each other and the community center staff. In contrast, the second community center was newer, and strong community ties had not been established. A substantial proportion of participants lived further away from the second community center. Such disparities likely explained the lower dropout rate at the first community center (n=12, 19%) as compared with the second community center (n=20, 32%, *P*=.11).

**Table 1:** Baseline characteristics.

Characteristic		Intervention group (n=55)	Control group (n=39)	*P* value
Age (years), mean (SD)		68.05 (6.56)	69.83 (5.86)	.13
Female, n (%)		39 (71)	29 (74)	.71
**Ethnicity, n (%)**		—^a^	—	.81
	Chinese	54 (98)	38 (97)	—
	Indian	1 (2)	1 (3)	—
**Highest education, n (%)**		—	—	.06
	Primary and below	15 (27)	6 (15)	—
	Secondary	23 (42)	20 (51)	—
	Postsecondary	14 (26)	10 (26)	—
	Tertiary and above	3 (6)	3 (8)	—
**Preferred language, n (%)**		—	—	.92
	English	26 (37)	18 (46)	—
	Mandarin	29 (53)	21 (54)	—
Modified Mini-Mental State Examination, mean (SD)		28.11 (1.78)	28.46 (1.52)	.33
Geriatric Depression Scale, mean (SD)		0.84 (1.23)	1.08 (1.51)	.36
Sedentary^b^, n (%)		21 (38)	11 (28)	.32
Class attendance, mean (SD)		17.24 (2.92)	14.28 (6.16)	.03
Adhered to randomization^c^, n (%)		41 (75)	32 (82)	.39
Dropouts, n (%)		14 (20)	17 (30)	.20
**RBANS^d^**		—	—	—
	Story memory subtest, mean (SD)	–0.17 (0.98)	0.36 (0.81)	.006
	Story recall subtest, mean (SD)	–0.08 (1.02)	0.33 (0.84)	.05

^a^Not applicable.

^b^Exercised less than once per week.

^c^Attended computerized cognitive training according to randomization sequence.

^d^RBANS: Repeatable Battery for the Assessment of Neuropsychological Status. Subtest scores were standardized within each assessment (ie, across groups) before being summed to derive the domain scores and a total score; baseline differences between the intervention and control group were examined using Pearson chi-square tests for categorical data, independent samples *t* tests for parametric data, and Mann-Whitney *U* tests for nonparametric data. There were no significant group differences for other outcome measures not reported in this table.

### Implementation Measures: Provider and Community Level

At the provider level, penetration into the target population was low while implementation fidelity was high. It was difficult to recruit the target population (ie, sedentary older adults) at community centers. Only 34% of participants exercised less than once per week. This eligibility criterion was relaxed, and research participation was extended to all regardless of exercise frequency. The maximum class size was 20. Each community center had sufficient demand to allow the formation of 3 to 4 CCT classes with 9 to 15 attendees each, ensuring efficient resource use. However, following dropouts, an English class was conducted for 3 participants who declined to join the bilingual class. CCT was implemented at both community centers with high fidelity. Class instructors adhered to class schedules and curriculum.

Facilitators and barriers to program delivery at the provider and community levels were resource availability, community partnerships, program demand, staff buy-in, and prior community implementation experience. At the provider level, the program provider secured a grant to cover some program costs (eg, instructor training and salary) while cofunding the remaining costs (eg, hardware, software). This allowed CCT to be offered at a subsidized rate of SGD $20, ensuring that it remained affordable to participants who were acquainted with subsidized fees for various community center programs. The program provider invested time and effort to establish community partnerships with the respective community centers. Participant recruitment was completed in 2 batches due to the resource-demanding nature of implementing multiple concurrent classes, recruitment issues, and interest in fine-tuning implementation for subsequent batches. Initially, the team sought to recruit 2 batches of participants from the same community center given the difficulty of securing implementation sites. However, subsequent recruitment efforts at the first community center revealed that demand for CCT has been exhausted. Engagement of the second community center delayed recruitment of the second batch by 4 months. The program provider pitched the CCT to 7 community centers and management committees before securing 2 implementation sites. The completion of this implementation at the community level could be attributed to extensive experience on the part of the community centers in delivering community-based programs. Both community centers leveraged existing administrative processes to promote the CCT and support class enrollment. They were also equipped with facilities needed for the CCT. Implementation was faster at the first community center due to better staff support and administrative processes.

### Participant Baseline Characteristics

Participants’ mean age was 68.8 (SD 6.3) years (Table 1). The majority were women, Chinese, and had at least secondary education. Dropouts were more likely to be sedentary compared with those who completed both assessments (*P*<.001). There were no baseline group differences in outcome variables in ITT analyses (Multimedia Appendix 2). In PP analyses, the intervention group had poorer baseline performance for RBANS story memory (*P*=.006) and story recall subtests (*P*=.05) compared with the control group (Table 1).

### Effectiveness Measures

#### ITT Analyses

The interaction term was not statistically significant for RBANS coding (*b*=–0.15, *P*=.06, 95% CI [–0.30 to 0.00]) and list recall subtests (*b*=–0.39, *P*=.05, 95% CI [–0.78 to 0.00]. For both RBANS subtests, performance in the intervention group declined post-CCT while the control group improved (Multimedia Appendix 3). The assessment term was significant for single task GVI (*b*=2.32, *P*=.03, 95% CI [0.30 to 4.35]) and RBANS list recognition subtest (*b*=–0.57, *P*=.009, 95% CI [–1.00 to –0.14]) but not statistically significant for RBANS picture naming subtest (*b*=0.36, *P*=.07, 95% CI [–0.3 to 0.74]). From baseline to follow-up, single task GVI and RBANS picture naming scores increased while performance in RBANS list recognition subtest deteriorated in both groups (Multimedia Appendix 3).

#### PP Analyses

**Figure 2:**
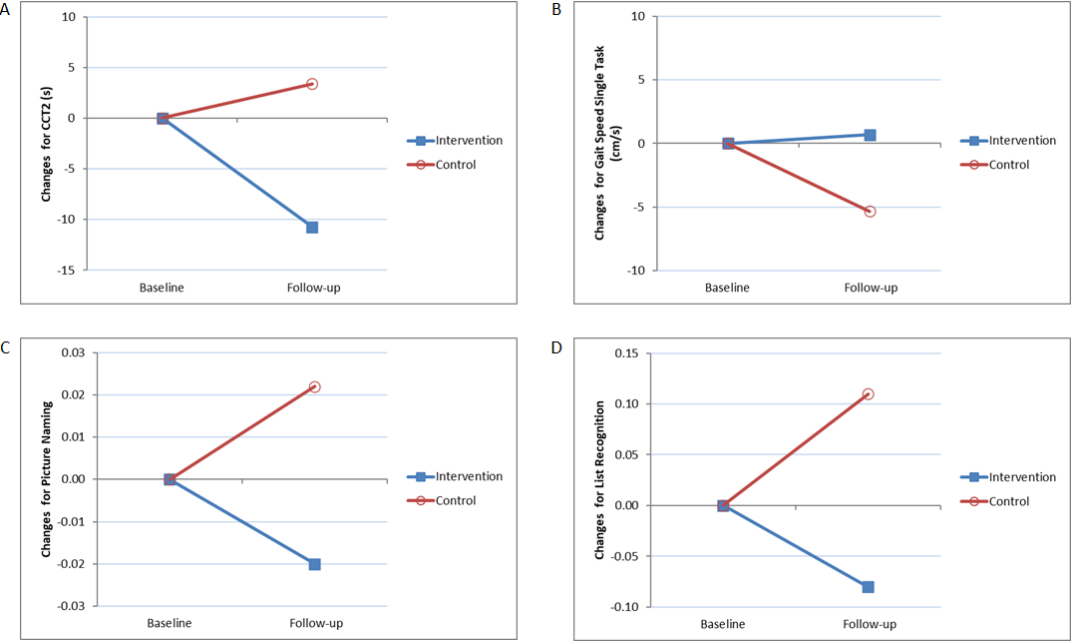
Results of per-protocol analyses.

The interaction term was significant for time taken to complete CTT-2 (*b*=–13.5, *P*=.01, 95% CI [–23.95 to –3.14], Cohen *d* effect size = 0.285) and not statistically significant for single task gait speed (*b*=5.38, *P*=.06, 95% CI [–0.30 to 11.36]; Figure 2 and Table 2). The assessment term was significant for RBANS picture naming (*b*=0.43, *P*=.046, 95% CI [0.01 to 0.85]) and list recognition subtests (*b*=–0.54, *P*=.02, 95% CI [–1.00 to –0.08]). From baseline to follow-up, both groups’ RBANS picture naming performance improved while their RBANS list recognition performance deteriorated (Figure 2 and Table 2). Effect sizes of the changes between baseline and follow-up in the intervention and control groups are presented in Multimedia Appendix 4 and Multimedia Appendix 5, respectively.

**Table 2:** Means and standard deviations for effectiveness measures.

Effectiveness measures		Baseline assessment^a^, mean (SD)			Follow-up assessment, mean (SD)	
		Intervention group (n=55)	Control group (n=39)	Intervention group (n=55)		Control group (n=39)
Time taken for Color Trails Test Part 2, seconds (n=91)^b^		124.43 (39.91)	111.99 (29.89)	113.69 (35.20)		115.38 (30.63)
**RBANS^c^ subtests^d^**						
	List learning	–0.04 (1.08)	0.06 (0.89)	–0.08 (1.03)		0.11 (0.96)
	Story memory	–0.24 (1.04)	0.33 (0.85)	–0.17 (1.16)		0.24 (0.66)
	Figure copy	–0.06 (1.08)	0.09 (0.89)	0.05 (1.09)		–0.07 (0.87)
	Line orientation	–0.13 (1.05)	0.18 (0.91)	–0.02 (1.08)		0.03 (0.89)
	Picture naming	–0.07 (1.04)	0.09 (0.95)	–0.09 (1.13)		0.11 (0.77)
	Semantic fluency	–0.13 (0.96)	0.18 (1.03)	–0.02 (1.04)		0.03 (0.95)
	Digit span	–0.13 (1.00)	0.18 (0.99)	–0.10 (0.92)		0.14 (1.10)
	Coding	–0.09 (1.09)	0.13 (0.86)	–0.09 (1.11)		0.12 (0.82)
	List recall	–0.05 (1.10)	–0.08 (0.85)	–0.07 (1.16)		0.10 (0.71)
	List recognition	–0.09 (1.16)	0.13 (0.72)	–0.17 (1.20)		0.24 (0.54)
	Story recall	–0.18 (1.05)	0.24 (0.87)	–0.18 (1.13)		0.25 (0.72)
	Figure recall	–0.06 (1.04)	0.08 (0.94)	0.06 (1.10)		–0.08 (0.84)
**RBANS domains^e^**						
	Immediate memory	–0.28 (1.80)	0.39 (1.44)	–0.25 (1.92)		0.35 (1.21)
	Visuospatial	–0.02 (1.70)	0.27 (1.35)	0.03 (1.77)		–0.05 (1.43)
	Language	–0.20 (1.54)	0.28 (1.63)	–0.10 (1.85)		0.15 (1.39)
	Attention	–0.22 (1.55)	0.30 (1.44)	–0.18 (1.64)		0.26 (1.62)
	Delayed memory	–0.28 (3.62)	0.39 (2.35)	–0.36 (3.81)		0.51 (1.74)
RBANS total score^e^		–1.15 (7.97)	1.62 (5.77)	–0.86 (9.08)		1.21 (5.00)
Berg Balance Scale (n=93)		52.75 (4.66)	54.61 (1.53)	53.27 (4.00)		54.08 (2.52)
**Gait speed^f^**						
	Single task (cm/s; n=92)	101.60 (22.60)	105.26 (20.41)	102.29 (22.36)		99.92 (19.03)
	Dual task (cm/s; n=91)	70.28 (23.50)	71.00 (25.75)	70.77 (22.29)		66.12 (20.44)
	Dual task cost (n=90)	–32.88 (14.16)	–32.53 (19.66)	–30.33 (19.85)		–34.12 (14.52)
**GVI^g^ (n=90)**						
	Single task	88.49 (6.43)	88.60 (6.29)	89.31 (5.62)		91.27 (6.59)
	Dual task	84.71 (13.14)	86.48 (15.64)	85.12 (13.02)		85.47 (12.59)

^a^Baseline differences between the intervention and control group were examined using Pearson chi-square tests for categorical data, independent samples *t* tests for parametric data, and Mann-Whitney *U* tests for nonparametric data.

^b^Time taken for Color Trails Test Part 2 was missing for 7 participants who exceeded the maximum time provided during 1 or more assessments.

^c^RBANS: Repeatable Battery for the Assessment of Neuropsychological Status.

^d^RBANS subtest scores were standardized within each assessment (ie, across groups) prior to analyses.

^e^RBANS domain scores and total score were derived by summing standardized subtest scores.

^f^Physical outcome measures were missing for selected participants for the following reasons: did not complete physical assessment at baseline (n=2), lack of eligible trials (eg, not performing serial subtraction during dual task condition, taking too few steps per trial, <3 trials for GVI derivation; n=3).

^g^GVI: Gait Variability Index.

## Discussion

### Principal Findings

In this single-blind RCT in 94 healthy community-dwelling adults, there were improvements in attention and executive function. We showed that it was feasible to implement community-based CCT in community centers to promote cognitive improvements in healthy older adults. Implementation measures revealed high reach and moderate adherence but poor penetration of sedentary older adults. Most community center users were physically and socially active. Participants paid for and attended 80% of classes. Attendance was higher in the intervention group than the control group. However, 25% dropped out and 22% did not adhere to randomization. Implementation was facilitated by CCT trainers’ in-class guidance and encouragement, participant proximity to and strong community ties at implementation sites, resource availability, strong community partnerships, staff buy-in, and prior program implementation experience.

### Implementation of Community-Based CCT Program

It is feasible to implement CCT at community centers for healthy older adults. At the participant level, there was good program reception and adherence. Participants were interested in CCT despite relative unfamiliarity with the technology. Reach was high (81%), and participants were willing to pay SGD $20 for the CCT, increasing the economic viability of CCT implementation in community centers provided it is subsidized as with other community center programs. Program interest and adherence could be attributed to in-class guidance, timely technical support from the program provider, and participants’ higher educational attainment [24]. Modest adherence was observed despite participants’ busy schedules. Dropout rate was 25%, largely due to busy schedules, loss of interest in research participation or CCT, and loss to follow-up. Class attendance was 80%, with higher attendance in the intervention group. In other local studies with community-based interventions, dropout and attendance rates were 2% to 11% and 79% to 95%, respectively [25-27]. The relatively higher dropout rate in this study was likely due to participant characteristics. The current sample consisted of healthy physically and socially active older adults already occupied with various community center programs or commitments. Future studies can include additional measures to boost adherence (eg, follow-up with participants who miss 2 consecutive CCT classes, maintain regular contact with the control group through monthly health talks). Another area for further study pertains to trade-offs regarding CCT frequency and duration, adherence, and effectiveness.

At the provider level, there was high fidelity to program implementation but low penetration into the target population. The program provider successfully delivered the program without deviating from planned schedules and curriculum. Instructors were well trained and well received. Participants cited trainers’ in-class guidance and encouragement as motivation to continue with CCT despite increasing gaming complexity. Penetration into the sedentary older population was low; only 34% exercised less than once per week. Different sites and recruitment strategies (eg, targeted outreach at senior activity centers) will be needed to reach out to sedentary older adults. When this eligibility criterion was relaxed, there was sufficient demand at each community center to allow formation of classes with 9 to 15 participants each. This ensured efficient resource use given a maximum class size of 20. Thereafter, demand at each community center is likely to be geared toward more advanced CCT to promote continuity and maintenance among experienced attendees. The feasibility and efficacy of advanced CCT requires further research.

Facilitators and barriers to CCT implementation at the participant, provider, and community level were identified. Participant adherence was boosted by CCT trainers’ in-class guidance, proximity to implementation sites, and strong community ties. Participants in the first community center lived nearer to the community center and were close-knit. Provider and community implementation success depended on resource availability, community partnerships, staff buy-in, and prior program implementation expertise. CCT could be offered at a subsidized rate with grant support and cofunding by the CCT provider. In community centers, CCT may not be feasible without subsidized class fees as community center-goers are acquainted with subsidized fees for community center programs. Lower demand and smaller class sizes would negate the minimal economy of scale CCT providers need. Successful implementation was also contingent on community partnerships between the program provider and implementation sites. Identifying suitable community centers required substantial time and effort from the program provider. The difficulty of establishing new partnerships delayed recruitment of the second batch of participants by 4 months. Within community centers, implementation success was associated with prior experience in delivering community-based programs and staff buy-in. Implementation was better executed at the first community center due to better staff support and administrative processes. Future implementers should consider population characteristics, program demand, prior program implementation experience, staff buy-in, and existing administrative processes and facilities when shortlisting implementation sites.

### Effectiveness of CCT for Healthy Older Adults

CCT improved executive function and attention in agreement with previous reports [8,14,15]. CCT’s impact on memory should be a subject of future implementation studies given previous reports of memory improvements [8,14,15].

In this study, CCT’s modest efficacy may be attributed to participants’ relatively high educational and physical activity levels. Participants were more educated than the average Singaporean older adult [25], and a substantial proportion were enrolled in various community center programs, including fitness classes and enrichment lessons. This might explain why high scores and even ceiling effects were observed for various RBANS subtests and the Berg Balance Scale at baseline. Participants had less room for cognitive improvements, with benefits observed only for attention and executive function.

We found that there was no significant improvement in gait speed from baseline to follow-up in the intervention group, while gait speed deteriorated in the control group. In prior studies [9-12], gait and balance improvements were more pronounced in sedentary older adults with low gait speed (ie, <1 m/s). In our study, 66.0% of the sample exercised at least once a week, a level higher than the average Singaporean older adult [28]. Low gait speed was also not observed in 61.3% of the sample at baseline. This suggests future investigation given gait improvements uncovered in prior studies [9-12]. More sensitive effectiveness measures and a larger sample size might be needed to detect the small effect size of CCT in healthy high-functioning older adults.

Given that 22% did not adhere to randomization, it was unsurprising that results for ITT and PP analyses differed. Of the 22%, 64% crossed over from their assigned control group to the intervention group. This predominant direction of switch likely explained unexpected findings in ITT analyses. From baseline to follow-up, the control group improved in RBANS coding and list recall subtests while performance in the intervention group declined. ITT analyses uncovered no baseline group differences across outcome measures while PP analyses found that the control group performed better in RBANS story memory and story recall subtests at baseline. The lack of adherence to randomization likely led to a situation where the control group had more individuals with better memory for narratives. More weight was given to PP analyses as it was difficult to interpret ITT analyses given the substantial number of individuals who did not adhere to randomization.

### Strengths and Limitations

CCT was implemented with high fidelity to planned class schedule and curriculum. In-class guidance and timely technical support mitigated technological barriers faced by older adults and likely boosted receptiveness to CCT. Attendance was comparable to other local studies with community-based interventions [25-27]. However, dropout rate was higher (25%) even though participants paid a subsidized fee to attend. The target population (ie, sedentary healthy older adults) could not be recruited at community centers. Instead, the inclusion criterion was relaxed to include older adults regardless of their exercise frequency. This, together with participants’ high educational attainment and participation in various community center programs, likely limited CCT’s effectiveness. We encountered difficulty recruiting enough sedentary older adults. In addition, the dropout rates were highest in the older adult group. This may suggest that the implementation in its current form is not suitable for the population at the community centers. Online implementation option of group training should be considered in the future, especially in the context of the COVID-19 pandemic. The convenience of online training may address adherence, group training, and maintaining motivation while achieving necessary social distancing. Nevertheless, the study remains informative given that the sample might be more representative of CCT attendees, especially in community-based or commercial settings. It also provides useful information on suitable healthy aging programs that can be implemented on a larger scale within communities.

Outcomes measures could be improved upon. The adapted RBANS and CTT-2 were not well validated for Singaporean older adults. Norms were available for Form A of both tests for Chinese Singaporean older adults [21]. Except for the digit span subtest, the English and Mandarin versions of RBANS Form A were equivalent in this population [29]. However, RBANS and CTT-2 Form B were modified for this study and have not been validated previously. RBANS was selected as the primary effectiveness measure as it was one of the few validated tools with parallel forms that assessed various cognitive domains. Implementation could have been evaluated more rigorously via standardized feedback questionnaires and semistructured interviews.

### Conclusion

In conclusion, this study showed that it was feasible to implement community-based CCT in community centers, and the CCT program resulted in modest cognitive improvements among healthy Singaporean older adults. With the study being conducted in a real-world setting, the challenges and necessary adjustments and adaptations made is common with such implementations, but it allows for lessons to be learned and documented for improvement. CCT can be implemented with relatively good uptake and adherence in community centers but at a subsidized fee as with other community center programs. Implementers should consider participants’ proximity to implementation sites, strength of community ties, resource availability, staff buy-in, and prior program implementation expertise when shortlisting implementation sites. Future studies can explore the effectiveness of community based CCT for older adults who are sedentary or at risk of cognitive impairment.

## Appendix

Multimedia Appendix 1 Implementation measures.

Multimedia Appendix 2 Baseline characteristics based on intention-to-treat analysis.

Multimedia Appendix 3 Effectiveness measures (intention-to-treat analysis)—means and standard deviations.

Multimedia Appendix 4 Effect sizes at baseline and follow-up in the intervention group.

Multimedia Appendix 5 Effect sizes at baseline and follow-up in the control group.

Multimedia Appendix 6 CONSORT-EHEALTH checklist (V 1.6.1).

## Abbreviations

CCT: computerized cognitive training
CTT-2: Color Trails Test 2
GVI: Gait Variability Index
ITT: intention-to-treat
PP: per protocol
RBANS: Repeatable Battery for the Assessment of Neuropsychological Status
RCT: randomized controlled trial
