# Supplementary material for: Evaluation of the Implementation and Effectiveness of Community-Based Brain-Computer Interface Cognitive Group Training in Healthy Community-Dwelling Older Adults: Randomized Controlled Implementation Trial
Source: JMIR Form Res. 2021 Apr 27;5(4):e25462. doi: 10.2196/25462 (PMC8114157; doi:10.2196/25462)
Supplement: Multimedia Appendix 1 [file formative_v5i4e25462_app1.docx]

Multimedia Appendix: Implementation Measures

| Measure | Description | Data |
| --- | --- | --- |
|  |  |  |
| **Participant-Level** |  |  |
| Reach | Proportion of eligible participants enrolled in the study | Percentage of eligible participants who enrolled in the study after screening |
| Adherence | Adherence to randomization | Percentage of participants who attended CCT classes randomly assigned to them |
|  | Dropouts | Percentage of participants who were classified as dropouts |
|  | Class attendance | Average CCT class attendance |
| Facilitators &  Barriers | Facilitators & barriers to participation | Through observations and as communicated through informal feedback |
|  |  |  |
| **Provider-Level** |  |  |
| Penetration | Penetration into target population | Percentage of participants who are sedentary |
| Implementation | Class size | Final class size |
|  | Adherence to planned class schedules & curriculum | Percentage of deviations from planned class schedules & curriculum |
| Facilitators &  Barriers | Facilitators & barriers to program delivery for Neeuro | Through observations and as communicated during meetings and feedback sessions |
|  |  |  |
| **Community-Level** |  |  |
| Facilitators &  Barriers | Facilitators & barriers to program delivery at community centers | Through observations and as communicated during meetings and feedback sessions |
