# Supplementary material for: Evaluation of the Implementation and Effectiveness of Community-Based Brain-Computer Interface Cognitive Group Training in Healthy Community-Dwelling Older Adults: Randomized Controlled Implementation Trial
Source: JMIR Form Res. 2021 Apr 27;5(4):e25462. doi: 10.2196/25462 (PMC8114157; doi:10.2196/25462)
Supplement: Multimedia Appendix 2 [file formative_v5i4e25462_app2.docx]

Multimedia Appendix: Baseline characteristics based on Intention-to-Treat Analysis

|  | Intention-to-Treat Analysis | | |
| --- | --- | --- | --- |
| Variables (N (%) or Mean (SD)) | Intervention Group (N = 48) | Control Group  (N = 46) | *P*-value |
|  |  |  |  |
| Age (at screening) | 69.09 (6.39) | 68.47 (6.27) | .60 |
| Female | 35 (72.9%) | 33 (71.7%) | .89 |
| Ethnicity |  |  |  |
| Chinese | 47 (97.9%) | 45 (97.8%) |  |
| Indian | 1 (2.1%) | 1 (2.2%) | .97 |
| Highest Education |  |  |  |
| Primary & Below | 10 (20.8%) | 11 (23.9%) |  |
| Secondary | 22 (45.8%) | 21 (45.7%) |  |
| Post-Secondary | 15 (21.3%) | 9 (19.6%) |  |
| Tertiary & Above | 1 (2.1%) | 5 (10.9%) | .24 |
| Preferred Language |  |  |  |
| English | 31 (50.8%) | 26 (40.6%) |  |
| Mandarin | 30 (49.2%) | 38 (59.4%) | .25 |
| Modified Mini-Mental State Examination (MMSE) | 28.38 (1.57) | 28.13 (1.80) | .62 |
| Geriatric Depression Scale (GDS) | 0.938 (1.24) | 0.935 (1.47) | .79 |
| Sedentary^a^ | 17 (35.4%) | 15 (32.6%) | .77 |
| Class Attendance | 17.35 (2.86) | 14.61 (5.85) | .02 |
| Adhered to Randomization^b^ | 41 (85.4%) | 32 (69.6%) | .06 |
| Dropouts | 13 (21.3%) | 18 (28.1%) | .37 |
| RBANS^c^ |  |  |  |
| Story Memory Subtest | -0.09 (1.08) | 0.09 (0.91) | .37 |
| Story Recall Subtest | -0.13 (1.04) | 0.14 (0.95) | .20 |

^a^Exercised less than once per week.

^b^Attended computerized cognitive training according to randomization sequence.

^c^RBANS subtest scores were standardized within each assessment (i.e., across groups) before being summed to derive the domain scores and a total score; Baseline differences between the intervention and control group were examined using Pearson’s chi-square tests for categorical data, independent samples t-test for parametric data, and Mann-Whitney U test for non-parametric data. There were no significant group differences for other outcome measures not reported in this table.
