# Supplementary material for: Evaluation of the Implementation and Effectiveness of Community-Based Brain-Computer Interface Cognitive Group Training in Healthy Community-Dwelling Older Adults: Randomized Controlled Implementation Trial
Source: JMIR Form Res. 2021 Apr 27;5(4):e25462. doi: 10.2196/25462 (PMC8114157; doi:10.2196/25462)
Supplement: Multimedia Appendix 3 [file formative_v5i4e25462_app3.docx]

Multimedia Appendix: Means and standard deviation for effectiveness measures (Intention-to-Treat Analysis)

|  | Intention-to-Treat Analysis | | | | |
| --- | --- | --- | --- | --- | --- |
|  | Baseline Assessment^a^ | | Follow-Up Assessment | |  |
| Effectiveness Measures,  Mean (SD) | Intervention Group  (N = 48) | Control Group  (N = 46) | Intervention Group  (N = 48) | Control  Group  (N = 46) |  |
|  |  |  |  |  |  |
| Time Taken for Color Trails Test Part 2, seconds  (N = 91)^b^ | 119.61 (38.87) | 118.60 (34.03) | 114.53 (33.74) | 114.30 (32.93) |  |
| RBANS Subtests^c^ |  |  |  |  |  |
| List Learning | -0.11 (1.09) | 0.12 (0.89) | -0.12 (1.02) | 0.13 (0.97) |  |
| Story Memory | -0.09 (1.08) | 0.09 (0.91) | -0.09 (1.14) | 0.09 (0.83) |  |
| Figure Copy | -0.03 (1.00) | 0.03 (1.01) | 0.01 (1.09) | -0.01 (0.91) |  |
| Line Orientation | 0.02 (1.02) | -0.03 (0.99) | -0.03 (1.04) | 0.03 (0.97) |  |
| Picture Naming | 0.03 (0.99) | -0.03 (1.02) | 0.00 (1.09) | 0.00 (0.91) |  |
| Semantic Fluency | -0.11 (1.00) | 0.11 (1.00) | -0.15 (1.02) | 0.16 (0.97) |  |
| Digit Span | -0.16 (0.99) | 0.16 (0.99) | -0.13 (0.95) | 0.14 (1.04) |  |
| Coding | 0.02 (0.98) | -0.02 (1.03) | -0.05 (0.99) | 0.05 (1.02) |  |
| List Recall | -0.04 (1.14) | 0.04 (0.84) | -0.19 (1.13) | 0.19 (0.81) |  |
| List Recognition | -0.14 (1.21) | 0.14 (0.70) | -0.20 (1.18) | 0.21 (0.72) |  |
| Story Recall | -0.13 (1.04) | 0.14 (0.95) | -0.16 (1.14) | 0.16 (0.80) |  |
| Figure Recall | 0.06 (1.06) | -0.06 (0.94) | 0.02 (1.07) | -0.02 (0.93) |  |
| RBANS Domains^d^ |  |  |  |  |  |
| Immediate Memory | -0.20 (1.84) | 0.21 (1.50) | -0.21 (1.85) | 0.22 (1.47) |  |
| Visuospatial | -0.01 (1.62) | 0.01 (1.54) | -0.02 (1.66) | 0.02 (1.62) |  |
| Language | -0.08 (1.53) | 0.08 (1.66) | -0.16 (1.77) | 0.16 (1.56) |  |
| Attention | -0.14 (1.52) | 0.15 (1.52) | -0.18 (1.54) | 0.19 (1.72) |  |
| Delayed Memory | -0.25 (3.70) | 0.26 (2.49) | -0.52 (3.65) | 0.54 (2.42) |  |
| RBANS Total Score^d^ | -0.68 (8.14) | 0.70 (6.18) | -1.09 (8.36) | 1.14 (6.83) |  |
| Berg Balance Scale  (N = 93) | 52.68 (4.68) | 54.35 (2.42) | 53.11 (4.15) | 54.11 (2.58) |  |
| Gait Speed^e^ |  |  |  |  |  |
| Single Task  (cm/s; N = 92) | 103.32 (23.04) | 102.91 (20.49) | 101.79 (22.58) | 100.84 (19.47) |  |
| Dual Task  (cm/s; N = 91) | 70.78 (25.63) | 70.37 (23.16) | 68.28 (20.62) | 69.49 (22.71) |  |
| Dual Task Cost  (N = 90) | -33.47 (16.45) | -32.00 (16.78) | -32.33 (19.94) | -21.55 (15.72) |  |
| GVI  (N = 90) |  |  |  |  |  |
| Single Task | 88.79 (6.88) | 88.29 (5.81) | 89.29 (6.23) | 90.99 (5.89) |  |
| Dual Task | 86.35 (13.85) | 84.49 (14.51) | 85.70 (13.57) | 84.81 (12.08) |  |

^a^Baseline differences between the intervention and control group were examined using Pearson’s chi-square tests for categorical data, independent samples t-test for parametric data, and Mann-Whitney U test for non-parametric data.

^b^Time taken for Color Trails Test Part 2 was missing for 7 participants who exceeded the maximum time provided during 1 or more assessments.

^c^RBANS subtest scores were standardized within each assessment (i.e., across groups) prior to analyzes.

^d^RBANS domain scores and total score were derived by summing standardized subtest scores.

^e^Physical outcome measures were missing for selected participants for the following reasons: did not complete physical assessment at baseline (N = 2), lack of eligible trials (e.g., not performing serial subtraction during dual task condition, taking too few steps per trial, < 3 trials for GVI derivation; N = 3).
