# Supplementary material for: Evaluation of the Implementation and Effectiveness of Community-Based Brain-Computer Interface Cognitive Group Training in Healthy Community-Dwelling Older Adults: Randomized Controlled Implementation Trial
Source: JMIR Form Res. 2021 Apr 27;5(4):e25462. doi: 10.2196/25462 (PMC8114157; doi:10.2196/25462)
Supplement: Multimedia Appendix 4 [file formative_v5i4e25462_app4.docx]

Multimedia Appendix: Effect sizes at baseline and at follow-up in the intervention group

|  | Intention-to-Treat Analysis | | | |  |  |  | Per-Protocol Analysis | | | |  |  |  |
| --- | --- | --- | --- | --- | --- | --- | --- | --- | --- | --- | --- | --- | --- | --- |
|  | Baseline Assessment | | Follow-Up Assessment | |  |  |  | Baseline Assessment | | Follow-Up Assessment | |  |  |  |
| Effectiveness Measures,  mean (SD) | Intervention Group | | Intervention Group | |  |  |  | Intervention Group | | Intervention Group | |  |  |  |
|  | N = | 48 | N = | 48 |  |  |  | N = | 55 | N = | 55 |  |  |  |
|  | Mean | SD | Mean | SD | Cohen's *d* | Glass's *delta* | Hedges' *g* | Mean | SD | Mean | SD | Cohen's *d* | Glass's *delta* | Hedges' *g* |
|  |  |  |  |  |  |  |  |  |  |  |  |  |  |  |
| Time Taken for CCT2, seconds | 119.61 | 38.87 | 114.53 | 33.74 | 0.140 | 0.131 | 0.140 | 124.43 | 39.91 | 113.69 | 35.20 | 0.285 | 0.269 | 0.285 |
| (N = 91) |  |  |  |  |  |  |  |  |  |  |  |  |  |  |
| RBANS Subtests |  |  |  |  |  |  |  |  |  |  |  |  |  |  |
| (N = 94) |  |  |  |  |  |  |  |  |  |  |  |  |  |  |
| List Learning | -0.11 | 1.09 | -0.12 | 1.02 | 0.009 | 0.009 | 0.009 | -0.04 | 1.08 | -0.08 | 1.03 | 0.038 | 0.037 | 0.038 |
| Story Memory | -0.09 | 1.08 | -0.09 | 1.14 | 0.000 | 0.000 | 0.000 | -0.24 | 1.04 | -0.17 | 1.16 | 0.064 | 0.067 | 0.064 |
| Figure Copy | -0.03 | 1.00 | 0.01 | 1.09 | 0.038 | 0.040 | 0.038 | -0.06 | 1.08 | 0.05 | 1.09 | 0.101 | 0.102 | 0.101 |
| Line  Orientation | 0.02 | 1.02 | -0.03 | 1.04 | 0.049 | 0.049 | 0.049 | -0.13 | 1.05 | -0.02 | 1.08 | 0.103 | 0.105 | 0.103 |
| Picture Naming | 0.03 | 0.99 | 0.00 | 1.09 | 0.029 | 0.030 | 0.029 | -0.07 | 1.04 | -0.09 | 1.13 | 0.018 | 0.019 | 0.018 |
| Semantic Fluency | -0.11 | 1.00 | -0.15 | 1.02 | 0.040 | 0.040 | 0.040 | -0.13 | 0.96 | -0.02 | 1.04 | 0.110 | 0.115 | 0.110 |
| Digit Span | -0.16 | 0.99 | -0.13 | 0.95 | 0.031 | 0.030 | 0.031 | -0.13 | 1.00 | -0.10 | 0.92 | 0.031 | 0.030 | 0.031 |
| Coding | 0.02 | 0.98 | -0.05 | 0.99 | 0.071 | 0.071 | 0.071 | -0.09 | 1.09 | -0.09 | 1.11 | 0.000 | 0.000 | 0.000 |
| List Recall | -0.04 | 1.14 | -0.19 | 1.13 | 0.132 | 0.132 | 0.132 | -0.05 | 1.10 | -0.07 | 1.16 | 0.018 | 0.018 | 0.018 |
| List Recognition | -0.14 | 1.21 | -0.20 | 1.18 | 0.050 | 0.050 | 0.050 | -0.09 | 1.16 | -0.17 | 1.20 | 0.068 | 0.069 | 0.068 |
| Story Recall | -0.13 | 1.04 | -0.16 | 1.14 | 0.027 | 0.029 | 0.027 | -0.18 | 1.05 | -0.18 | 1.13 | 0.000 | 0.000 | 0.000 |
| Figure Recall | 0.06 | 1.06 | 0.02 | 1.07 | 0.038 | 0.038 | 0.038 | -0.06 | 1.04 | 0.06 | 1.10 | 0.112 | 0.115 | 0.112 |
| RBANS Domains |  |  |  |  |  |  |  |  |  |  |  |  |  |  |
| (N =94) |  |  |  |  |  |  |  |  |  |  |  |  |  |  |
| Immediate  Memory | -0.20 | 1.84 | -0.21 | 1.85 | 0.005 | 0.005 | 0.005 | -0.28 | 1.80 | -0.25 | 1.92 | 0.016 | 0.017 | 0.016 |
| Visuospatial | -0.01 | 1.62 | -0.02 | 1.66 | 0.006 | 0.006 | 0.006 | -0.02 | 1.70 | 0.03 | 1.77 | 0.028 | 0.029 | 0.028 |
| Language | -0.08 | 1.53 | -0.16 | 1.77 | 0.048 | 0.052 | 0.048 | -0.20 | 1.54 | -0.10 | 1.85 | 0.059 | 0.065 | 0.059 |
| Attention | -0.14 | 1.52 | -0.18 | 1.54 | 0.026 | 0.026 | 0.026 | -0.22 | 1.55 | -0.18 | 1.64 | 0.025 | 0.026 | 0.025 |
| Delayed Memory | -0.25 | 3.70 | -0.52 | 3.65 | 0.073 | 0.073 | 0.073 | -0.28 | 3.62 | -0.36 | 3.81 | 0.022 | 0.022 | 0.022 |
| RBANS Total Score (N = 94) | -0.68 | 8.14 | -1.09 | 8.36 | 0.050 | 0.050 | 0.050 | -1.15 | 7.97 | -0.86 | 9.08 | 0.034 | 0.036 | 0.034 |
| Berg Balance Scale | 52.68 | 4.68 | 53.11 | 4.15 | 0.097 | 0.092 | 0.097 | 52.75 | 4.66 | 53.27 | 4.00 | 0.120 | 0.112 | 0.120 |
| (N = 93) |  |  |  |  |  |  |  |  |  |  |  |  |  |  |
| Gait Speed |  |  |  |  |  |  |  |  |  |  |  |  |  |  |
| Single Task | 103.32 | 23.04 | 101.79 | 22.58 | 0.067 | 0.066 | 0.067 | 101.60 | 22.60 | 102.29 | 22.36 | 0.031 | 0.031 | 0.031 |
| (cm/s; N = 92) |  |  |  |  |  |  |  |  |  |  |  |  |  |  |
| Dual Task | 70.78 | 25.63 | 68.28 | 20.62 | 0.107 | 0.098 | 0.107 | 70.28 | 23.50 | 70.77 | 22.29 | 0.021 | 0.021 | 0.021 |
| (cm/s; N = 91) |  |  |  |  |  |  |  |  |  |  |  |  |  |  |
| Dual Task Cost | -33.47 | 16.45 | -32.33 | 19.94 | 0.062 | 0.069 | 0.062 | -32.88 | 14.16 | -30.33 | 19.85 | 0.148 | 0.180 | 0.148 |
| (N = 90) |  |  |  |  |  |  |  |  |  |  |  |  |  |  |
| GVI (N = 90) |  |  |  |  |  |  |  |  |  |  |  |  |  |  |
| Single Task | 88.79 | 6.88 | 89.29 | 6.23 | 0.076 | 0.073 | 0.076 | 88.49 | 6.43 | 89.31 | 5.62 | 0.136 | 0.128 | 0.136 |
| Dual Task | 86.35 | 13.85 | 85.70 | 13.57 | 0.047 | 0.047 | 0.047 | 84.71 | 13.14 | 85.12 | 13.02 | 0.031 | 0.031 | 0.031 |
