# Supplementary material for: Evaluation of the Implementation and Effectiveness of Community-Based Brain-Computer Interface Cognitive Group Training in Healthy Community-Dwelling Older Adults: Randomized Controlled Implementation Trial
Source: JMIR Form Res. 2021 Apr 27;5(4):e25462. doi: 10.2196/25462 (PMC8114157; doi:10.2196/25462)
Supplement: Multimedia Appendix 5 [file formative_v5i4e25462_app5.docx]

Multimedia Appendix: Effect sizes at baseline and at follow-up in the control group

|  | Intention-to-Treat Analysis | | | |  |  |  | Per-Protocol Analysis | | | |  |  |  |
| --- | --- | --- | --- | --- | --- | --- | --- | --- | --- | --- | --- | --- | --- | --- |
|  | Baseline Assessment | | Follow-Up Assessment | |  |  |  | Baseline Assessment | | Follow-Up Assessment | |  |  |  |
| Effectiveness Measures,  mean (SD) | Control Group | | Control Group | |  |  |  | Control Group | | Control Group | |  |  |  |
|  | N = | 46 | N = | 46 |  |  |  | N = | 39 | N = | 39 |  |  |  |
|  | Mean | SD | Mean | SD | Cohen's *d* | Glass's *delta* | Hedges' *g* | Mean | SD | Mean | SD | Cohen's *d* | Glass's *delta* | Hedges' *g* |
|  |  |  |  |  |  |  |  |  |  |  |  |  |  |  |
| Time Taken for CCT2, seconds | 118.60 | 34.03 | 114.30 | 32.93 | 0.128 | 0.126 | 0.128 | 111.99 | 29.89 | 115.38 | 30.63 | 0.112 | 0.113 | 0.112 |
| (N = 91) |  |  |  |  |  |  |  |  |  |  |  |  |  |  |
| RBANS Subtests |  |  |  |  |  |  |  |  |  |  |  |  |  |  |
| (N = 94) |  |  |  |  |  |  |  |  |  |  |  |  |  |  |
| List Learning | 0.12 | 0.89 | 0.13 | 0.97 | 0.011 | 0.011 | 0.011 | 0.06 | 0.89 | 0.11 | 0.96 | 0.054 | 0.056 | 0.054 |
| Story Memory | 0.09 | 0.91 | 0.09 | 0.83 | 0.000 | 0.000 | 0.000 | 0.33 | 0.85 | 0.24 | 0.66 | 0.118 | 0.106 | 0.118 |
| Figure Copy | 0.03 | 1.01 | -0.01 | 0.91 | 0.042 | 0.040 | 0.042 | 0.09 | 0.89 | -0.07 | 0.87 | 0.177 | 0.175 | 0.177 |
| Line  Orientation | -0.03 | 0.99 | 0.03 | 0.97 | 0.061 | 0.061 | 0.061 | 0.18 | 0.91 | 0.03 | 0.89 | 0.167 | 0.165 | 0.167 |
| Picture Naming | -0.03 | 1.02 | 0.00 | 0.91 | 0.031 | 0.029 | 0.031 | 0.09 | 0.95 | 0.11 | 0.77 | 0.025 | 0.023 | 0.025 |
| Semantic Fluency | 0.11 | 1.00 | 0.16 | 0.97 | 0.051 | 0.050 | 0.051 | 0.18 | 1.03 | 0.03 | 0.95 | 0.151 | 0.146 | 0.151 |
| Digit Span | 0.16 | 0.99 | 0.14 | 1.04 | 0.020 | 0.020 | 0.020 | 0.18 | 0.99 | 0.14 | 1.10 | 0.038 | 0.040 | 0.038 |
| Coding | -0.02 | 1.03 | 0.05 | 1.02 | 0.068 | 0.068 | 0.068 | 0.13 | 0.86 | 0.12 | 0.82 | 0.012 | 0.012 | 0.012 |
| List Recall | 0.04 | 0.84 | 0.19 | 0.81 | 0.182 | 0.179 | 0.182 | -0.08 | 0.85 | 0.10 | 0.71 | 0.230 | 0.212 | 0.230 |
| List Recognition | 0.14 | 0.70 | 0.21 | 0.72 | 0.099 | 0.100 | 0.099 | 0.13 | 0.72 | 0.24 | 0.54 | 0.173 | 0.153 | 0.173 |
| Story Recall | 0.14 | 0.95 | 0.16 | 0.80 | 0.023 | 0.021 | 0.023 | 0.24 | 0.87 | 0.25 | 0.72 | 0.013 | 0.011 | 0.013 |
| Figure Recall | -0.06 | 0.94 | -0.02 | 0.93 | 0.043 | 0.043 | 0.043 | 0.08 | 0.94 | -0.08 | 0.84 | 0.179 | 0.170 | 0.179 |
| RBANS Domains |  |  |  |  |  |  |  |  |  |  |  |  |  |  |
| (N =94) |  |  |  |  |  |  |  |  |  |  |  |  |  |  |
| Immediate  Memory | 0.21 | 1.50 | 0.22 | 1.47 | 0.007 | 0.007 | 0.007 | 0.39 | 1.44 | 0.35 | 1.21 | 0.030 | 0.028 | 0.030 |
| Visuospatial | 0.01 | 1.54 | 0.02 | 1.62 | 0.006 | 0.006 | 0.006 | 0.27 | 1.35 | -0.05 | 1.43 | 0.230 | 0.237 | 0.230 |
| Language | 0.08 | 1.66 | 0.16 | 1.56 | 0.050 | 0.048 | 0.050 | 0.28 | 1.63 | 0.15 | 1.39 | 0.086 | 0.080 | 0.086 |
| Attention | 0.15 | 1.52 | 0.19 | 1.72 | 0.025 | 0.026 | 0.025 | 0.30 | 1.44 | 0.26 | 1.62 | 0.026 | 0.028 | 0.026 |
| Delayed Memory | 0.26 | 2.49 | 0.54 | 2.42 | 0.114 | 0.112 | 0.114 | 0.39 | 2.35 | 0.51 | 1.74 | 0.058 | 0.051 | 0.058 |
| RBANS Total Score (N = 94) | 0.70 | 6.18 | 1.14 | 6.83 | 0.068 | 0.071 | 0.068 | 1.62 | 5.77 | 1.21 | 5.00 | 0.076 | 0.071 | 0.076 |
| Berg Balance Scale | 54.35 | 2.42 | 54.11 | 2.58 | 0.096 | 0.099 | 0.096 | 54.61 | 1.53 | 54.08 | 2.52 | 0.254 | 0.346 | 0.254 |
| (N = 93) |  |  |  |  |  |  |  |  |  |  |  |  |  |  |
| Gait Speed |  |  |  |  |  |  |  |  |  |  |  |  |  |  |
| Single Task | 102.91 | 20.49 | 100.84 | 19.47 | 0.104 | 0.101 | 0.104 | 105.26 | 20.41 | 99.92 | 19.03 | 0.271 | 0.262 | 0.271 |
| (cm/s; N = 92) |  |  |  |  |  |  |  |  |  |  |  |  |  |  |
| Dual Task | 70.37 | 23.16 | 69.49 | 22.71 | 0.038 | 0.038 | 0.038 | 71.00 | 25.75 | 66.12 | 20.44 | 0.210 | 0.190 | 0.210 |
| (cm/s; N = 91) |  |  |  |  |  |  |  |  |  |  |  |  |  |  |
| Dual Task Cost | -32.00 | 16.78 | -21.55 | 15.72 | 0.643 | 0.623 | 0.643 | -32.53 | 19.66 | -34.12 | 14.52 | 0.092 | 0.081 | 0.092 |
| (N = 90) |  |  |  |  |  |  |  |  |  |  |  |  |  |  |
| GVI (N = 90) |  |  |  |  |  |  |  |  |  |  |  |  |  |  |
| Single Task | 88.29 | 5.81 | 90.99 | 5.89 | 0.462 | 0.465 | 0.462 | 88.60 | 6.29 | 91.27 | 6.59 | 0.414 | 0.424 | 0.414 |
| Dual Task | 84.49 | 14.51 | 84.81 | 12.08 | 0.024 | 0.022 | 0.024 | 86.48 | 15.64 | 85.47 | 12.59 | 0.071 | 0.065 | 0.071 |
